# Dietary patterns and their associations with obesity among 4–9-year-old children in the United Arab Emirates: A cross-sectional study

**DOI:** 10.1371/journal.pone.0352032

**Published:** 2026-07-13

**Authors:** Farah Naja, Nada Abbas, Leila Itani, Fatima Al Zahraa Chokor, Leila Cheikh Ismail, Ayesha S. Al Dhaheri, Lynda O’Neill, Samer Kharroubi, Habiba I. Ali, Maysm N. Mohamad, Nahla Hwalla, Lara Nasreddine

**Affiliations:** 1 Department of Clinical Nutrition and Dietetics, College of Health Sciences, Research Institute of Medical and Health Sciences, University of Sharjah, Sharjah, United Arab Emirates; 2 Department of Nutrition and Dietetics, Faculty of Health Sciences, Beirut Arab University, Beirut, Lebanon; 3 Department of Public Health, College of Health Sciences, QU Health, Qatar University, Doha, Qatar; 4 Department of Nutrition and Health, College of Medicine and Health Sciences, United Arab Emirates University, Al Ain, United Arab Emirates; 5 Nestlé Institute of Health Sciences, Nestlé Research, Société des Produits Nestlé S.A., Lausanne, Switzerland; 6 Department of Nutrition and Food Sciences, Faculty of Agricultural and Food Sciences, American University of Beirut, Beirut, Lebanon; University of Petra (UOP), JORDAN

## Abstract

Dietary patterns constitute a modifiable lifestyle exposure, affecting obesity among children. This study aimed to identify and characterize dietary patterns amongst young school-aged children in the UAE and investigate their association with obesity. A cross-sectional survey of 4–9-year-old children (n = 426) was conducted in the 3 major Emirates of the UAE. Socio-demographic characteristics and anthropometric measurements were obtained. Dietary assessment was performed using the 24-hour recall method. Derivation of dietary patterns was carried out using factor analysis. Of the study sample, 19.5% were overweight/at risk of overweight, and 5% were obese. Two main dietary patterns were derived: “Healthy Pattern”, marked by higher consumption of fruits and vegetables, olive oil, and whole grains, and “Western Pattern,” characterized by higher intakes of fast food, salty snacks, sweets, and sugar-sweetened beverages. The Healthy Pattern demonstrated negative correlations with total energy, saturated and trans-fat intake, and a positive correlation with dietary fiber intake. The Western Pattern was positively correlated with proteins, cholesterol, saturated and trans-fat intake, and negatively correlated with dietary fiber intake. Higher scores of the Healthy Pattern were associated with lower BMI-for-age Z scores. These findings provide evidence for the development of culture-specific nutrition programs for curbing the obesity epidemic amongst children in the UAE.

## Introduction

Childhood obesity remains a pressing public health issue on a global scale, with its prevalence steadily increasing across various regions of the world [1,2]. Available data suggest that the Eastern Mediterranean Region (EMR) harbors one of the highest rates of childhood overweight and obesity [3], particularly in countries of the Gulf Cooperation Council [3]. The early school age has been reported as a key stage for the development of obesity [4], as it is often associated with significant changes in the child’s lifestyle. For primary school-children, the family’s daily routine undergoes fundamental restructuring and the demands on children typically increase, such as the requirement to be punctual and to be sitting most of the day [5]. The dietary habits of children also change in this life stage with a higher reliance on snacks and foods available within the school setting [4].

Pediatric obesity may lead to adverse physical and psychological effects that appear in childhood and tend to track into adulthood [6]. Cardiometabolic complications such as impaired glucose regulation, dyslipidemia, high blood pressure and metabolic syndrome are amongst the well-described short-term health consequences of childhood obesity [7]. Pediatric obesity is also likely to persist into the adult life stage, increasing the risk for chronic diseases such as type 2 diabetes, cardiovascular diseases, and specific cancers in the long-term [8].

The etiology of childhood obesity is multifactorial and often involves a complex interplay between genetic, environmental and behavioral factors [9]. A positive energy balance, which tends to result from the regular consumption of energy-dense, high fat and/or high sugar foods, is considered amongst the main causes of obesity [10,11]. In this respect, nutrition research has initially focused on examining individual nutrients or foods in relation to obesity, but more recently, attention has shifted towards identifying and analyzing holistic dietary patterns [10,11]. This approach offers a comprehensive understanding of the overall diet, encompassing the combination of various food groups and taking into consideration the complex interactions that may exist between the different foods and nutrients [8,12]. Studies conducted amongst school-aged children from various parts of the world have suggested that certain dietary patterns characterized by frequent consumption of refined cereals, energy dense, micronutrient poor foods such as fast foods and sweets correlate with higher body mass index (BMI) and adiposity [13,14]. For instance, a study conducted amongst 6–16 year old children from various European countries showed that the majority of children who were overweight and obese (39% and 56%, respectively) were allocated to the “Refined Cereals” cluster [15]. A study conducted in China amongst 6–17 year old children [16] showed that the Western dietary pattern was significantly associated with an increased risk of obesity (OR: 1.49; 95% 1.21, 1.84) after adjusting for potential confounders, whereas the traditional Chinese pattern was not associated with obesity in this age group. Studies conducted in the Eastern Mediterranean Region are scarce. In Iran, Bahreynian et al (2013) reported that lower adherence to the “sweet and dairy” pattern was associated with lower BMI among girls aged 7–11 years (OR = 0.42, CI = 0.21, 0.85), whereas no significant associations were found for boys [17].

In the Gulf Council Coperation countries, studies investigating dietary patterns as a determinant of childhood obesity are lacking, despite the high burden of obesity in the region. Particularly, data stemming from a study conducted in 3 Emirates of the UAE showed a high prevalence of child obesity in school-aged children (14.3% in 4–13 year olds) [18]. It is in this context that this study was conducted, with the aim of 1) deriving and characterizing dietary patterns amongst young school-aged children aged 4–9 years in the UAE; 2) investigating the association of the identified dietary patterns with obesity in this age group and 3) evaluating the association of the derived patterns with energy, macro- and selected micronutrients intakes. The results of this study can inform tailored interventions aimed at combating childhood obesity and fostering a culture of healthy eating practices among children.

## Materials and methods

### Study design

Data for this study were drawn from a previous large-scale cross-sectional survey aimed at investigating food consumption patterns, dietary intakes and food sources of nutrients in children, aged 4 to 12.9 years in the UAE. The original survey was conducted in line with the protocol of the Global Kids Nutrition and Health Study (KNHS) [19]. In the UAE, the KNHS took place in the three largest Emirates of the country: Abu Dhabi, Dubai, and Sharjah. Recruitment of children was carried out using a stratified random cluster sampling frame, within which the emirates constituted the strata. Schools-including preschools, considered as clusters, were randomly selected from each stratum.

### Study population

At the selected schools, all children meeting the inclusion criteria were invited to participate. Inclusion criteria for study participation required children to fall within the age range of 4 to 12.9 years, not to have any inborn errors of metabolism, chronic medical conditions, or physical disabilities that might impact their dietary behaviors or anthropometric measurements. Arab non-nationals residing in the UAE for less than three years, as well as those with mothers under 18 years of age, were excluded. The minimum residency period of three years was set to ensure that the children’s eating habits observed in the study reflected the typical dietary patterns prevalent in the country. Further details about the sampling used in the UAE KNHS are described elsewhere [20]. To enable comparison, a sample of Arab non-national children were also recruited at a ratio of 2:1 (national to non-national). Non-Arabs were excluded from the study due to notable variations in dietary habits, cuisine, and cultural foods between Arabs (including UAE nationals) and non-Arabs. The total sample of the original survey of children was 646 (431 national participants and 215 non-national participants). Ethical approval for the study protocol was obtained from the Institutional Review Boards (IRB) of the American University of Beirut (AUB), the United Arab Emirates University (UAEU), Dubai Health Authority (DHA), UAE Ministry of Health and Prevention (MOHAP), Ministry of Education in the United Arab Emirates (MOE), and the University of Sharjah (UOS). As compensation for their time spent participating in the study, caregivers were offered a 15-dollar book voucher.

For this study, analysis was restricted to children aged 4–9 years, as this age window represents the early school years, a particularly critical developmental period during which dietary habits are being established and the risk of obesity onset is notably heightened, enabling a focus on a developmentally homogeneous population with comparable dietary behaviors. For this study, data of children aged 4–9 years were used (n = 426). The availability of a sample size of 426 allowed the detection of an effect size (regression coefficient beta) of 4%, at a power of 80% and a 95% confidence interval [21].

### Study protocol

Data collection took place between June 4, 2019 and March 27, 2020. Trained research nutritionists conducted data collection within the preschool or school settings. The administration offices of the selected schools were approached to introduce the study and to plan for data collection at their premises. Information sheets explaining the study objectives and protocols were distributed to all eligible students to be shared with their parents. Parents who agreed to participate signed the information sheet and returned it with the child. Upon receipt, the research team contacted the parent and scheduled a meeting at the school’s clinic. The meeting was requested with the main caregiver of the child, which in the case of this study, was the mother for all children. During this meeting, details about the study protocol and objectives were shared with the parents, who once agreed, signed a written consent. Data collection was conducted in a one-to-one interview with the parent, in the presence of the child.

### Data collection

Data collection consisted of a multicomponent questionnaire as well as an assessment of anthropometric measurements for the child and the mother. Data collection took around 30 minutes. The questionnaire addressed socio-economic characteristics, lifestyle habits of the child (sleep and physical activity), in addition to dietary intake. The questions included in the questionnaire were formulated in line with the global KNHS protocol [19]. Adaptations to the local Emirati context were carried out by a panel of experts involved in this research which included a dietitian, public health epidemiologist, and a social worker.

### Sociodemographic and lifestyle characteristics

This section collected information regarding the age and sex of the child, the age of the mother, and the education and employment status of both the mother and the father. Questions inquiring about the number of family members and the number of rooms in the house were used to derive the crowding index (number of family members/ number of rooms). This index is used as a proxy for socioeconomic status with higher values indicating lower socioeconomic status [22]. In this section, questions regarding taking vitamins (yes/no), being a picky eater (yes/no), and the presence of a live-in helper (yes/no) were included. The lifestyle characteristics included sleep and physical activity. Mothers were asked to report the total number of hours their child slept during the last 24 hours, including naps. Using the American Association of Pediatric recommendations, children were grouped as either met or did not meet sleep recommendations (age 4–5 years: 10–13 hours; age 6–9 years: 9–12 hours) [23]. For physical activity, mothers completed the Youth Physical Activity Questionnaire (Y-PAQ). The latter addressed the frequency and duration of various types of physical activity of the child over the last 7 days [24]. The World Health Organization (WHO) recommendations for physical activity were used to group children as either met or did not meet the requirements for physical activity (age 3–4: at least 180 minutes in a variety of types of physical activities at any intensity, of which at least 60 minutes is moderate- to vigorous-intensity physical activity, spread throughout the day; age 5–9 years: at least an average of 60 minutes per day of moderate-to-vigorous intensity, mostly aerobic, physical activity, across the week) [25].

### Dietary intake

The multiple pass 24-Hour Recall (24-HR) method was used to collect information regarding dietary intake, with mothers as proxies. The 24-HR, developed by the United States Department of Agriculture (USDA), follows a structured five-step approach to the collection of dietary intake data, minimizing recall bias and enhancing the accuracy of the reported food consumption [26]. The first step of the 24-HR method involves a quick run-through of the foods and beverages consumed by the child in the previous 24 hours. This initial pass aimed to create a broad outline of the child’s dietary intake, capturing major meals and snacks consumed. The second step examines specific details of the meal consumed, such as the type of food, preparation methods, brand names, and any accompanying condiments or beverages, probing for any additional foods that might have been overlooked in the initial recall. The third step focuses on portion size estimation. Mothers were offered the choice to estimate portions using standard household measures, such as teaspoons, tablespoons, and cups, which are commonly found in most kitchens. Research assistants carried models of these household measures to assist the participants in specifying their portion size. In addition, mothers were presented with the two-dimensional visual chart that depicts various food portions. This chart served as a helpful reference tool, enabling mothers to visually compare their food portions to those depicted in the chart, thereby aiding in more accurate estimations [27]. Dietary data analysis was performed using the Nutritionist Pro Software (v 5.1.0, 2014, First Data Bank, Nutritionist Pro, Axxya Systems, San Bruno, CA, USA).Using local recipes, mixed dishes from the UAE were inputted into the software individually as standalone food items [28]. The dietary intake analysis relied on various food composition databases such as the USDA database, food composition tables specific to the Middle East [29], and where applicable, nutritional details derived from product packaging and online platforms.

### Anthropometric assessments

The weight and height of the child and the mother were assessed using standardized protocols [30]. Height was measured without shoes, using a stadiometer (Seca 217, Hamburg, Germany) and measurements of weight were obtained to the nearest 0.1 kg with the participant wearing light clothing using a clinical balance (Seca 874, Hamburg, Germany). For the mother, the BMI was calculated by dividing the weight (in kg) by height in meters squared. For the child, the anthropometric measurements were evaluated using WHO AnthroPlus software [31]. The WHO Child Growth Criteria, based on the BMI for Age Z-Score (BAZ), were used to identify children within the wasting, normal, overweight and obese categories, as follows: children aged 4–5 years, wasting <−2; normal status −2 ≤ BAZ ≤ +1; possible risk of overweight +1 ≤ BAZ ≤ +2; overweight +2 ≤ BAZ ≤ +3; and obese as BAZ > + 3; children aged 5–9 years, wasting <−2; normal status −2 ≤ BAZ ≤ +1; overweight status +1 ≤ BAZ ≤ +2; and obese status of BAZ > +2 [32].

### Data Analysis

The statistical analysis was conducted using SPSS statistical software version 26. Dietary patterns were derived using Factor Analysis (FA). Food items available from the 24 HR data were grouped into 13 food groups (Supplementary S1 Table). The grouping of the food items was carried out based on their culinary usage and shared nutrient composition. The total gram intake per day from all items within each food group was used to calculate the consumption from that group. The Kaiser–Meyer–Olkin (KMO) test (0.536) and the χ2 Bartlett test of sphericity (P  <  0.001) indicated that the data was suitable for FA. The number of factors to retain in the FA was based on the Kaiser’s criterion (eigenvalue greater than 1) and the interpretability of the resulting factors. An orthogonal transformation (varimax) of the retained factors was then implemented to maximize the variance of the squared loadings and enhance the interpretability of the dietary patterns. These derived patterns were labeled according to the food groups exhibiting factor loadings greater than 0.4. Factor scores were computed using a regression approach, assigning each participant a score for each identified pattern. These scores represented the extent to which an individual adhered to a specific dietary pattern, with higher scores indicating a greater level of adherence.

Baseline characteristics of the study population were described, by BAZ categories. Continuous variables were expressed as means ± standard deviation (SD), while categorical variables were presented as frequencies and percentages. Significances were assessed using the independent sample t-test for continuous variables and chi-square for categorical variables. To examine the relationship between the derived dietary patterns and BAZ, both simple and multiple linear regression analyses were performed, with the latter incorporating variables that exhibited a p-value < 0.2 in the simple regression model. Beta coefficients, 95% confidence intervals (CI), and corresponding p-values were reported. Pearson correlation coefficients were calculated to examine the association between the derived dietary patterns and energy, macro- and selected micronutrient intake. A p-value less than 0.05 was considered statistically significant.

## Results

The baseline characteristics of the study population, categorized by BAZ, are summarized in Table 1. The study included 426 children (wasted: 10.3%, normal weight: 65.2%, overweight (including at risk of overweight): 19.5%, obese: 5%). The mean age of the children was 5.72 years (±1.37), almost evenly distributed between males (47.9%) and females (52.1%). Emirati nationality constituted the majority (64.3%) of the study population. Maternal age (mean 35.82 ± 6.09) and BMI (mean 28.12 ± 5.08) were significantly associated with children’s BAZ categories (p-values 0.027 and 0.038, respectively). Notably, mothers of children in the “obese” BAZ category were older and had a higher BMI compared to other categories. About half of the parents had a university education or higher (48.6% for mothers and 51.4% for fathers). Employment rates were 90% for fathers and 46.2% for mothers. Concerning living conditions, 64.9% of children were born in families with a crowding index of <2 persons per room. Supplement use was reported by 23.9%. Adherence to physical activity and sleep recommendations was observed among 40.8% and 67.6%, respectively. Picky eating behavior was prevalent (52.2%), and significantly associated with BAZ (p-value <0.001), with a higher proportion of picky eaters in the “obese” BAZ category. The presence of a live-in helper was reported by 60.6% of participants.

**Table 1:** Baseline characteristics of the study population, by BAZ categories (n = 426).

	Total(N = 426)	BAZ categories^*^				
		Wasted (n = 44)	Normal (n = 278)	At risk of being overweight/ overweight (n = 83)	Obese(n = 21)	p-value
**Child’s age (years)**	5.72 ± 1.37	5.64 ± 1.4	5.65 ± 1.32	5.92 ± 1.51	6 ± 1.34	0.964
**Child’s sex**						0.105
Male	204(47.9)	24(47.9)	24(54.5)	127(54.5)	127(45.7)	
Female	222(52.1)	20(52.1)	20(45.5)	151(45.5)	151(54.3)	
**Child’s nationality**						0.285
Emirati	274(64.3)	33(64.3)	33(75)	179(75)	179(64.4)	
Not Emirati	152(35.7)	11(35.7)	11(25)	99(25)	99(35.6)	
**Mother’s age(years)**	35.82 ± 6.09	33.68 ± 5.39	35.97 ± 6.19	35.73 ± 6.17	39.06 ± 4.12	0.027^†^
**Mother’s BMI (kg/m**^**2**^)	28.12 ± 5.08	26.05 ± 3.85	27.74 ± 4.83	29.69 ± 5.25	31.5 ± 6.93	0.038^†^
**Mother’s education**						0.342
Lower education/ intermediate/ high school	219(51.4)	28(51.4)	28(63.6)	137(63.6)	137(49.3)	
University or higher	207(48.6)	16(48.6)	16(36.4)	141(36.4)	141(50.7)	
**Father’s education**						0.043^†^
Lower education/ intermediate/ high school	205(48.6)	28(48.6)	28(63.6)	121(63.6)	121(43.8)	
University or higher	217(51.4)	16(51.4)	16(36.4)	155(36.4)	155(56.2)	
**Mother working**						0.731
Not working	229(53.8)	24(53.8)	24(54.5)	149(54.5)	149(53.6)	
Working	197(46.2)	20(46.2)	20(45.5)	129(45.5)	129(46.4)	
**Father working**						0.335
Not working	27(6.4)	1(6.4)	1(2.3)	16(2.3)	16(5.8)	
Working	395(93.6)	43(93.6)	43(97.7)	260(97.7)	260(94.2)	
**Crowding index**						0.351
<2 person per room	274(64.9)	23(64.9)	23(53.5)	180(53.5)	180(65.2)	
≥ 2 person per room	148(35.1)	20(35.1)	20(46.5)	96(46.5)	96(34.8)	
**Supplementation (vitamins, minerals or both)**						0.166
No	324(76.1)	33(76.1)	33(75)	206(75)	206(74.1)	
Yes	102(23.9)	11(23.9)	11(25)	72(25)	72(25.9)	
**Meet weekly physical activity recommendations**						0.982
No	252(59.2)	25(59.2)	25(56.8)	165(56.8)	165(59.4)	
Yes	174(40.8)	19(40.8)	19(43.2)	113(43.2)	113(40.6)	
**Meet daily sleep recommendation**						0.153
No	138(32.4)	8(32.4)	8(18.2)	93(18.2)	93(33.5)	
Yes	288(67.6)	36(67.6)	36(81.8)	185(81.8)	185(66.5)	
**Picky eater**						<0.001^†^
No	202(47.8)	18(47.8)	18(40.9)	119(40.9)	119(43.3)	
Yes	221(52.2)	26(52.2)	26(59.1)	156(59.1)	156(56.7)	
**Live-in helper**						0.902
No	168(39.4)	16(39.4)	16(36.4)	108(36.4)	108(38.8)	
Yes	258(60.6)	28(60.6)	28(63.6)	170(63.6)	170(61.2)	

Abbreviations: BAZ, BMI-for-age z score; BMI, body mass index.

Categorical variables are presented as frequency and percentage n (%), while continuous variables

are presented as mean ± standard deviation.

* Wasting was defined as BAZ <−2, normal as −2 ≤ BAZ≤+1, at risk of

being overweight as+1 ≤ BAZ≤+2, overweight as+2 ≤ BAZ≤+3, and obesity as BAZ > +3

† Indicates a significant p-value at alpha level 0.05.

Table 2 provides a description of the two identified dietary patterns and their corresponding food groups, as derived from FA: “Western Dietary Pattern” and the “Healthy Dietary Pattern.” The suitability of the data for factor analysis was supported by the KMO test value of 0.536 and Bartlett’s test of sphericity, which was statistically significant (χ², *p* < 0.001). The number of factors retained was determined based on Kaiser’s criterion, using eigenvalues greater than 1, as well as the interpretability of the resulting factors. The two retained dietary patterns explained a total of 24.76% of the variance in the data, with the Western Dietary Pattern explaining 12.6% and the Healthy Dietary Pattern explaining 12.2%. The Western Dietary Pattern was characterized by higher intakes of animal proteins, refined grains, and starchy vegetables, as indicated by positive factor loadings of these food groups. Additionally, this pattern was negatively associated with mixed traditional dishes, legumes, and nuts. In contrast, the Healthy Dietary Pattern was marked by higher consumption of fruits and vegetables, olive and vegetable oils, whole grains, and milk and dairy products. Fast food, salty snacks, sweets and sugar-sweetened beverages had negative loadings on this dietary pattern.

**Table 2:** Food groups intakes and their corresponding factor loading according to the two derived dietary patterns.

	Mean intake in grams	Standard Error	% Contribution to total Kcal	Standard Error	Factor loading Western Pattern	Factor loading Healthy Dietary Pattern
**Animal proteins (beef, lamb, chicken, fish and eggs)**	78.55	3.82	11.12	0.53	**0.673**	0.051
**Refined grains**	160.26	7.44	22.35	0.77	**0.672**	−0.171
**Mixed traditional dishes**	165.35	13.19	13.47	0.8	**−0.546**	−0.04
**Starchy vegetables**	7.14	1.59	0.54	0.13	**0.297**	0.159
**Legumes and nuts**	16.44	2.81	2.03	0.33	−0.157	−0.003
**Fruits and vegetables**	158.62	8.96	6.57	0.39	0.104	**0.585**
**Sweets and sugar sweetened beverages**	221.33	8.96	18.32	0.72	−0.176	**−0.543**
**Olive and vegetable oils**	1.49	0.3	0.38	0.09	−0.081	**0.455**
**Fast food**	28.82	3	6.31	0.65	−0.249	**−0.437**
**Salty snacks**	8.95	0.94	3.09	0.29	0.172	**−0.409**
**Whole grains**	14.53	1.48	2.83	0.29	−0.21	**0.397**
**Milk and dairy products**	204.56	9.19	12.99	0.51	0.09	**0.208**
**Variance explained**					12.6%	12.2%

**Fig 1:**
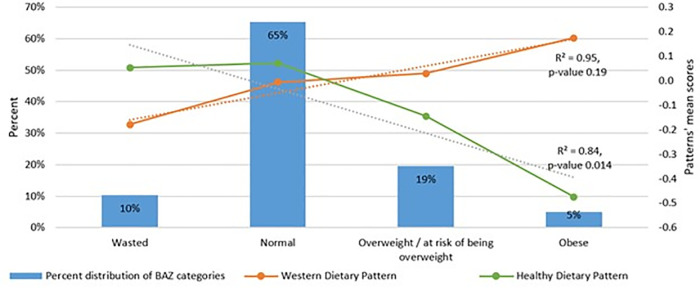
Mean scores of the Western and Healthy dietary patterns based on BAZ among the study population. Legend: Abbreviations: BAZ: BMI for Age Z-Score.

Table 3 shows the association of the derived dietary pattern with BAZ. While no significant association was found between the Western Dietary Pattern and BAZ, higher scores of the Healthy Dietary Pattern were associated with lower BAZ, in both the crude and adjusted models. The adjusted model included variables associated with BAZ at *p* < 0.20 in Table 2, namely child’s sex, mother’s age, mother’s BMI, father’s education, supplementation, meeting daily sleep recommendations, and picky eating. The adjusted beta coefficient in the multiple linear regression analyses was −0.07; 95% CI: −0.15 to −0.01; p-value = 0.043 (Table 3). Fig 1 presents the mean scores of both dietary patterns by weight categories. Children in the normal weight category exhibited higher mean scores of Healthy Dietary Pattern compared to those in the overweight or obese categories. In turn, those in the overweight and obese categories had higher scores of the Western Dietary Pattern than children in the normal BAZ category (Fig 1).

**Table 3:** Linear regression analyses for the associations of the derived dietary patterns with BAZ in the study population.

	beta	95% CI	p-value
**Western dietary pattern**			
Crude model	0.04	(−0.02; 0.11)	0.19
Adjusted model*	0	(−0.08; 0.08)	>0.999
**Healthy dietary pattern**			
Crude model	−0.08	(−0.15; −0.02)	**0.014**
Adjusted model*	−0.07	(−0.15; −0.01)	**0.043**

Abbreviations: BAZ, BMI-for-age z score; BMI, body mass index; CI, confidence interval.

* Variables included in the adjusted model include those who had a p-value <0.2 in Table 2: the child’s sex, mother’s age and BMI, father’s education, supplementation, meeting daily sleep recommendations, and picky eating.

**Fig 2:**
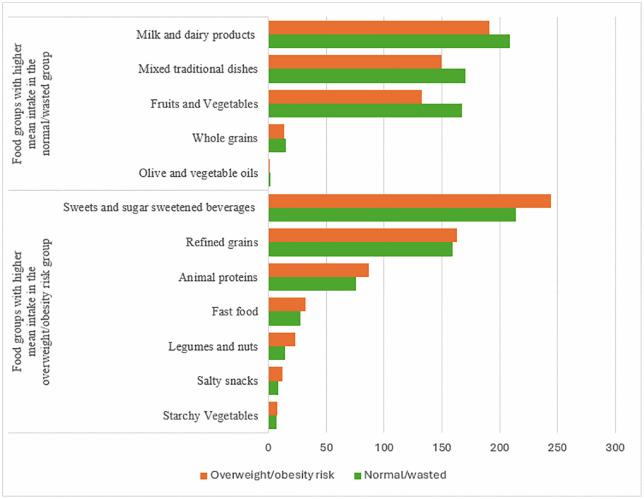
Mean intake of food groups in grams stratified by BAZ status. Legend: Abbreviations: BAZ: BMI for Age Z-Score.

The mean intakes of the various food groups stratified by BAZ status are presented in Fig 2. As depicted in this figure, higher intakes of SSB, refined grains, animal proteins, fast food, legume and nuts, salty snacks were reported by children with overweight and obesity. On the other hand, these children were reported to consume less milk and dairy products, mixed dishes, fruits and vegetables and whole grains.

Table 4 presents the relationships between the factor scores of the derived dietary patterns and the energy, macro- and selected micronutrient intake among the study participants. Significant positive correlations were observed between the scores of the Western Dietary Pattern and carbohydrates (r = 0.12), proteins (r = 0.32), cholesterol (r = 0.25), saturated and trans-fat ((r = 0.26), and iron (r = 0.23) intakes. On the other hand, the scores of this pattern were negatively associated with fat (r = −1.99), monounsaturated fat (r = −0.2), polyunsaturated fat (r = −0.24), dietary fiber (r = −0.19), and calcium (r = −0.11). In contrast, the Healthy Dietary Pattern demonstrated negative correlations with total energy intake (r = 0.2), carbohydrates (r = −0.17), fat (r = −0.18), saturated and trans-fat (r = −0.33), monounsaturated fat (r = −0.13), polyunsaturated fat (r = −0.2), and iron (r = −0.1). A positive correlation was observed between the Healthy Dietary Pattern and dietary fiber (r = 0.19).

**Table 4:** Pearson correation coefficients for the association of the scores of the derived dietary patterns with energy, macro- and selected micronutrients intake, among the study participants^*†^.

	Western dietary pattern	Healthy dietary pattern
Energy (total Kcal)	0.04	−0.20*
Carbohydrates (% total Kcal)	0.12*	−0.17*
Proteins (% total Kcal)	0.32**	−0.02
Fat (% total Kcal)	−1.99**	−0.18**
Cholesterol (mg)	0.25**	0.04
Saturated and trans-fat (g)	0.26**	−0.33**
Monounsaturated fat (g)	−0.20**	−0.13**
Poly unsaturated fat (g)	−0.24**	−0.20**
Dietary fiber (g)	−0.19**	0.19**
Vitamin C (mg)	0.01	0.07
Calcium (mg)	−0.11*	0.03
Iron (mg)	0.23**	−0.10*

* Values are significant at p < 0.05; ** Values are significant at p < 0.01

† The correlation coefficients for energy, macro- and selected micronutrients with the Western pattern were found to be significantly different from those with the healthy pattern (p < 0.05 for all comparisons).

## Discussion

This study is the first to characterize dietary patterns amongst young school-aged children in the Gulf Council Cooperation and their association with overweight and obesity in this age group. Two main dietary patterns were identified: the “Western Pattern” and the “Healthy Pattern.” Higher scores of the “Healthy” pattern were associated with lower BAZ while no significant association was observed for the Western pattern. Together, the two patterns identified in this study explained 24.76% of the total variance in dietary intakes amongst 4–9-year-old children, which is within the range of variance reported in the literature [33,34]. It remains important to note that there remains a substantial 75% of dietary intake variance unexplained, highlighting the complexity and multifaceted nature of dietary habits in this young age group.

Several studies conducted on school-aged children have also identified two major dietary patterns in this age group, such as those conducted in China [16] and Lebanon [35]. In contrast, other studies have identified three dietary patterns in this population group, such as the “Healthy”, “Transitive” and “Western” dietary patterns in China [36], the “Healthy,” “Western,” and “Sweet-Dairy” in Iran [17] and the “Sweet & Fat”, “Refined Cereals”, and “Animal Products” in Europe (Hebestreit 2017). It is important to highlight that factor analysis is a data-driven approach and discrepancies in the dietary assessment methodologies, the types and number of food groupings and the statistical analysis techniques may explain the observed variability in the identified dietary patterns across the literature [37]. It is noteworthy that the dietary patterns described in our study have been also reported by a previous study conducted amongst Emirati adults [38], while noting that the latter had also identified a third pattern, namely the “Traditional Emirati Pattern”. This discrepancy in relation to the traditional pattern is not surprising given that adherence to traditional diets has been repeatedly shown to be higher in older age groups compared to children and adolescents, a phenomenon that may be linked to the nutrition transition [39]. In fact, amidst the globalization and Westernization of diets and lifestyles, children are always more prone to the adoption of new convenience food consumption habits that tend to increase their adherence to Western dietary patterns [39,40], while decreasing their adoption of traditional diets.

One of the main criticisms of the dietary pattern approach is the labelling of the factors (i.e., patterns), which may be based on the subjective interpretation of the investigators. In our study, the correlations of the patterns’ scores with energy and nutrients’ intakes allowed us to further characterize the factors and justify the labelling. Like most studies examining dietary patterns, the pattern associated with higher fiber intakes and lower saturated and trans-fat intakes was labeled, in our study, as the “Healthy” pattern, whereas the one associated with lower fiber and calcium intakes and higher saturated/trans-fat intakes, was depicted as the Western pattern [35,41,42].

In agreement with findings reported by studies conducted elsewhere [35,43], the Western Dietary Pattern was characterized by higher intakes of animal proteins, refined grains, and starchy vegetables, while being negatively associated with mixed traditional dishes, legumes, and nuts. On the other hand, the Healthy Dietary Pattern was characterized by higher consumption of fruits and vegetables, olive and vegetable oils, whole grains, and milk and dairy products, and negatively associated with fast food, salty snacks, sweets and sugar-sweetened beverages, a finding that is also in line with the literature [36,41]. The study findings showed that higher adherence to the Healthy dietary pattern was associated with lower BAZ, and that children with obesity had a significantly lower adherence to the Healthy pattern. These results are consistent with those reported from China by Shang et al (2012) who showed that, compared to children (6−13 years old) with the highest adherence to the healthy dietary pattern, the odds of obesity were significantly higher for children adhering to the transitive pattern (Odds ratio (OR): 1.11; 95%CI: 0.89–1.38) and the Western pattern (OR: 1.80; 95%CI: 1.15–2.81) [36]. Similarly, in a study conducted amongst 7−11 year old children in Iran, Bahreynian et al (2013) showed that compared to girls in the highest quartile of the healthy dietary pattern, those in the second quartile were more likely to have higher BMI (OR:2.23,95% CI: 1.003, 4.96) [17]. These observations may be explained by the combinations of foods and nutrients that characterize the healthy dietary pattern and which may play synergistic roles in preventing excessive weight gain. For instance, fruits, vegetables and whole grains contain dietary fiber, phytochemicals and other bioactive compounds that may modulate appetite hormones such as glucagon-like peptide (GLP-1) and peptide YY (PYY) [44], improve the gut microbiome [45], and enhance insulin sensitivity [46], all of which may decrease the risk of excessive weight gain [44]. Higher intakes of dairy products, another hallmark of the identified healthy dietary pattern, have been also previously linked to reduced obesity risk in children, an observation that may be explained by the independent or synergistic effects of calcium and dairy protein on the regulation of body fat, lipolysis, lipogenesis and thermogenesis [47,48]. Surprisingly, the healthy dietary pattern has loaded positively on “olive and vegetable oils” intake. Studies have suggested that, as compared to saturated fat, unsaturated fatty acids from olive oil and other vegetable oils can play a role in decreasing the risk of obesity in childhood [49]. In fact, unsaturated fatty acids, and particularly monounsaturated fats, may have an impact on thermogenesis, the lipolytic activity of the adipocyte and the modulation of appetite, which may contribute to the regulation of body weight in childhood [49]. In addition, acknowledging that higher intakes of dietary energy and total fat may contribute to creating a positive energy balance and increasing the risk of obesity [50], the fact that the Healthy dietary pattern was characterized by lower consumption of energy-dense foods (fast food, salty snacks, sweets and sugar-sweetened beverages), and lower energy and fat intakes may also explain the observed inverse association between adherence to the Healthy pattern and BAZ.

It is important to recognize that, in contrast to our results, other studies did not find any association between dietary patterns and pediatric overweight or obesity, highlighting the controversy in the literature [43]. For instance, while Choi et al (2011) [43] have identified three dietary patterns amongst 7–8 year old children in Korea (Korean, modified Western, and Western patterns), they showed that the odds of obesity did not differ between the three dietary patterns. In addition, unlike the findings reported by some other investigations [16], the association between the Western pattern scores and BAZ did not reach statistical significance in our study. These results may be explained by some of the characteristics of the identified Western pattern. In fact, although the Western pattern loaded positively on refined grains, starchy vegetables and animal-based products, it was not associated with higher intakes of sweets, sweetened beverages, fast food or total fat. This may have diluted the observed association between this pattern and BMI. It is important to acknowledge that the specific foods contributing to each pattern may vary between studies given the data-driven nature of the factor analysis [40]. In addition, the discrepancies between the various study findings may arise from differences in age-groups, selection of food groupings, number of retained patterns and their labels as well as the type of confounders the studies have adjusted for [51,52].

The findings of this study should be interpreted considering the following limitations. First, the three selected Emirates for this study collectively house 85% of the UAE’s population, and therefore the exclusion of the remaining 15% residing in the other four Emirates may constitute a limitation. Second, and importantly, the cross-sectional design of this study constitutes a methodological limitation with respect to causal inference. Because dietary intake and anthropometric outcomes were measured simultaneously at a single time point, it is not possible to establish the direction of any observed associations. Specifically, it cannot be determined whether adherence to the Healthy dietary pattern preceded and contributed to lower BMI-for-age Z-scores, or whether children’s weight status influenced their dietary choices. This is particularly relevant in the context of pediatric obesity research, where reverse causation cannot be excluded since parents of children with obesity may modify their child’s diet in response to weight concerns. Accordingly, the findings of this study should be interpreted as cross-sectional associations, and causal conclusions should be avoided. Prospective longitudinal studies, or ideally intervention trials, are needed to confirm the direction and causality of the associations observed in this study. The third limitation is possibly related to the factor analysis methodology, a data-driven approach which tends to characterize population-specific patterns [40]. Therefore, our results possibly represent patterns that are, in some features, specific to the pediatric population in the UAE. In addition, the use of factor analysis entails several arbitrary assumptions such as those related to the choice of food groupings, the number of identified factors as well as their labels [51,52]. However, to decrease subjectivity in these approaches, the food groupings adopted in this study were comparable to those described by others [16,17,43] and the selection of the retained factors was conducted after the evaluation of the screen plots and eigenvalues. Another limitation stems from the adoption of the 24-HR for dietary assessment, a method that may be prone to reporting bias by the caregivers [53]. Nevertheless, despite its reliance on memory and the potential day-to-day variation in consumption patterns, the 24-HR approach was found to accurately estimate dietary energy intakes at the population level [54]. Moreover, the adoption of the multiple pass approach when implementing the recalls may have helped in attenuating the potential limitations of the 24-HR [55]. Additionally, to reduce interviewer errors, the 24-HRs were administered by research nutritionists who underwent extensive training prior to the initiation of data collection. The nutritionists who performed the interviews were also trained to avoid verbal and non-verbal judgmental cues in order to reduce social desirability bias.

## Conclusions

In conclusion, this study identified two major dietary patterns among school-aged children in the UAE: the “Western Pattern” and the “Healthy Pattern,” with the latter being associated with lower risk of obesity in the study population. These findings should foster the development of culture-specific programs and interventions aimed at curbing the burden of pediatric overweight/obesity, which has been shown to be on the rise in the UAE [56]. Interventions promoting the adoption of heathier dietary patterns, characterized by higher intakes of fruits, vegetables, whole grains and dairy products, while being low in fast food, sweets, sweetened beverages and other energy-dense foods should be developed. In fact, it has been shown that the provision of dietary guidance using a dietary pattern approach tends to be clearer and easier to comprehend and implement than approaches focused on solely nutrients, and this may be specifically valid for children and their caregivers [51]. Recognizing that healthy eating patterns adopted in childhood may exert immediate nutritional benefits, while also potentially tracking into the adult years, interventions that aim at fostering and establishing healthier food consumption habits and dietary skills in childhood may modulate the individual’s lifetime risk for diet-related morbidity.

## Supporting information

S1 Table Description of food groups included in the PCA. (DOCX)

S1 Questionnaire Completed copy of PLOS’ questionnaire on “Inclusivity in global research”. (DOCX)

## Abbreviations

24-HR: 24-Hour Recall
AUB: American University of Beirut
BAZ: BMI for Age Z-Score
BMI: body mass index
CI: confidence interval
DHA: Dubai Health Authority
EMR: Eastern Mediterranean Region
FA: factor analysis
GLP-1: glucagon-like peptide
IRB: Institutional Review Boards
KMO: Kaiser–Meyer–Olkin
KNHS: Kids Nutrition and Health Study
MOE: Ministry of Education
MOHAP: Ministry of Health and Prevention
OR: odds ratio
PYY: peptide YY
SD: standard deviation
UAE: United Arab Emirates
UAEU: United Arab Emirates University
UOS: University of Sharjah
USDA: United States Department of Agriculture
WHO: World Health Organizaion
Y-PAQ: Youth Physical Activity Questionnaire
