# Correction: Dietary patterns and their associations with obesity among 4–9-year-old children in the United Arab Emirates: A cross-sectional study

**DOI:** 10.1371/journal.pone.0356616

**Published:** 2026-08-18

**Authors:** Farah Naja, Nada Abbas, Leila Itani, Fatima Al Zahraa Chokor, Leila Cheikh Ismail, Ayesha S. Al Dhaheri, Lynda O’Neill, Samer Kharroubi, Habiba I. Ali, Maysm N. Mohamad, Nahla Hwalla, Lara Nasreddine

The Funding statement for this article is incorrect. The correct Funding statement is as follows: The parent study, conducted in the UAE was funded by Feeding Infants and Toddlers Study – Kids Nutrition and Health Study’ (FITS-KNHS) in the UAE (Nestle Research Center) (grant #: 2017NH3). The present study, however, is based on a secondary analysis of the parent study data, and did not receive any funding. The funders had no role in study design, data collection and analysis, decision to publish, or preparation of the manuscript.
