# Supplementary material for: Dietary patterns and their associations with obesity among 4–9-year-old children in the United Arab Emirates: A cross-sectional study
Source: PLoS One. 2026 Jul 13;21(7):e0352032. doi: 10.1371/journal.pone.0352032 (PMC13362138; doi:10.1371/journal.pone.0352032)
Supplement: S1 Table — (DOCX) [file pone.0352032.s001.docx]

Table S1. Description of food groups included in the PCA

| **Food Group** | **Description** |
| --- | --- |
| Refined grains | Noodles; refined breakfast Cereals; Rice; Pasta; Arabic saj; Markouk; White bread; Paratha |
| Milk and dairy products | Cheese; Milk; Yogurt; Labneh; Formula Milk |
| Fast food | Hamburger; fried chicken, fast food meals with French Fries; Ketchup or Tomato Catsup; |
| Fruits and vegetables | Arugula; Broccoli; Carrots; Cucumber; Lettuce; Mushrooms; Onions; Pepper; Tomatoes; Salads (Fattoush, Tabbouleh, etc.); Apple; Banana; Blueberries; Figs; Grapes; Pomegranate; Watermelon; Dates |
| Olive and vegetable oils | Olive oil; Peanut Butter; Sesame Butter or Tahini; Olives |
| Mixed traditional dishes | Bamieh Bi Lahmeh (okra with meat); Daoud basha; Eggplant stuffed with tomato and meat (sheikh el mehshi - batinjan); Mloukhiye (Jew's mallow with chicken and beef); Vegetable stew; Fish and Vegetables; Tuna Salad; Gratin (pasta with bechamel sauce and chicken); Laban immo (shekrieh); Shish barak; Bamieh bil zeit; Vine leaves, stuffed with meat (warak enab); Cabbage stuffed with meat; Lamb Biryani; Makloubeh; Beef Biryani; Loubieh bil Zeit |
| Legumes and nuts | Beans; Lentils; Nuts (Almonds, Pistachio, Walnut, Peanuts) |
| Animal proteins | Chicken; Fish; Beef; Turkey; Eggs |
| Salty snacks | Chips; Crackers; Nachos; Popcorn |
| Sweets and sugar-sweetened beverages | Arabic Sweets (Halawa, Maamoul, Nammoura, Rice pudding, Sahlab, Aasidah, Loukmiyat, etc.); Tiramisu; Brownie, Prepared; Cake, Cheesecake, Ready to Eat; Jellybeans; Lollipop; Marshmallows; Honey; Caramel; Sugar; Candy Bar, Milk Chocolate; Biscuit; Muffins; Cookies; Sandwich Cookies; Nescafe; Juice Blends; Pineapple Juice; Soda; Milkshake; Chocolate Milk; Sweetened Condensed Milk; Milkshake; Crepes; Doughnut; Pancake; Waffle |
| Starchy vegetables | Potatoes; Corn; Peas, Beets, Sweet Potatoes |
| Whole grains | Whole Wheat Cereals; Oats; Whole Wheat Bread; Enriched Noodles |
