# Supplementary material for: Dietary patterns and their associations with obesity among 4–9-year-old children in the United Arab Emirates: A cross-sectional study
Source: PLoS One. 2026 Jul 13;21(7):e0352032. doi: 10.1371/journal.pone.0352032 (PMC13362138; doi:10.1371/journal.pone.0352032)
Supplement: S1 Questionnaire — (DOCX) [file pone.0352032.s002.docx]

Questionnaire S1. Inclusivity in global research

PLOS’ policy on inclusivity in global research aims to improve transparency in the reporting of research performed outside of researchers’ own country or community and ensures that PLOS publications reporting global research adhere to high standards for research ethics and authorship. Authors of relevant research articles may be asked to complete the questionnaire below, which outlines ethical, cultural, and scientific considerations specific to inclusivity in global research. This questionnaire may be requested when researchers have travelled to a different country to conduct research, if research uses samples collected in another country, research with Indigenous populations or their lands, or if research is on cultural artefacts. Researchers travelling to another country solely to use laboratory equipment will not normally be required to complete the questionnaire. However, the questionnaire can be requested at the journal’s discretion for any submission – if you have been requested to complete this questionnaire by the PLOS journal you submitted to, please do so.

Please complete the questionnaire below and include this as a Supporting Information file with your manuscript. Note that if your paper is accepted for publication, this checklist will be published with your article in the supporting information files. Please ensure that you reference the checklist in the main body of your manuscript. We suggest adding a subsection ‘Inclusivity in global research’ to your Methods section and adding the following sentence: “Additional information regarding the ethical, cultural, and scientific considerations specific to inclusivity in global research is included in the Supporting Information (SX Checklist)”

The questions have been designed to be applicable to a wide range of study types, and there are subsections for both human subjects research and non-human subjects research. If any of the questions are not relevant to your research please mark them as “N/A” as appropriate.

**Ethical considerations, permits and authorship**

*This section is applicable to all research types.*

**Provide details as to who granted permissions and/or consent for the study to take place in the Methods section of your manuscript. This should include the names of all ethics boards, governmental organizations, community leaders or other bodies that provided approval for the study. If individuals provided approval refer to these people by their role or title but do not list their name(s).**

Reported on page number: 119-123

**If there were any deviations from the study protocol after approval was obtained please provide details of these changes in the Methods section of your manuscript.**
**Did this study involve local collaborators that are residents of the country where the research was conducted or members of the community studied? If you do not have any authors from said communities, please provide an explanation for this below.**

Reported on page number: not applicable

*Yes. This study involved local collaborators who are residents of the United Arab Emirates and members of the communities studied. Local collaborators contributed to all stages of the research, including study design, data collection, and engagement with local communities. Researchers based in Lebanon were also involved in conceptualization, data cleaning, analysis, and interpretation, working collaboratively with UAE-based team members throughout the research process.*

Everyone listed as an author should meet PLOS’ criteria for authorship and all individuals who meet these criteria should be included in the author byline, rather than the acknowledgements. For further information please see the journal’s Authorship Policy.

**Human subjects research (e.g. health research, medical research, cross-cultural psychology)**

**Did you obtain written informed consent from a representative of the local community or region before the research took place? How did you establish who speaks for the community?** **Details of written informed consent obtained from study participants should be reported separately in the Methods section of your manuscript.**

*Formal written informed consent from a single community representative was not obtained, as the study did not involve a unified community governance structure requiring such approval. Instead, the research protocol underwent comprehensive ethical review and approval by multiple institutional and national bodies, including the Institutional Review Boards of the American University of Beirut, United Arab Emirates University, the Dubai Health Authority, the UAE Ministry of Health and Prevention, the Ministry of Education in the UAE, and the University of Sharjah. These approvals ensured that the study met all ethical, regulatory, and community engagement requirements within the UAE context. In addition, local collaborators were actively involved in all stages of the research, including data collection and interaction with participants, ensuring cultural and contextual appropriateness*

**How did members of the local community provide input on the aims of the research investigation, its methodology, and its anticipated outcome(s)? When engaging with the local community, how did you ensure that the informed consent documents and other materials could be understood by local stakeholders?**

*Local community input was incorporated through the active involvement of UAE-based collaborators who are familiar with the healthcare system and the communities studied. These collaborators contributed to refining the research aims to ensure relevance to local public health priorities, as well as to the development and adaptation of the study methodology, including recruitment strategies and data collection procedures. They also played a key role in interpreting findings within the appropriate cultural and healthcare context, ensuring that the anticipated outcomes were meaningful and applicable to the local setting.*

*To ensure that informed consent documents and study materials were understandable to participants, all materials were developed and reviewed by local collaborators and, where appropriate, provided in languages commonly spoken in the UAE (e.g., Arabic and/or English). The wording was adapted to be culturally appropriate and easily comprehensible. In addition, data collectors—who were locally based—were trained to clearly explain the study objectives, procedures, and participants’ rights, and to address any questions prior to obtaining informed consent.*

**Will the findings of the research be made available in an understandable format to stakeholders in the community where the study was conducted (e.g. via a presentation, summary report, copies of publications, etc.)? Please provide details of how this will be achieved.**

*Yes. The findings of this research have been and will continue to be disseminated to stakeholders in the UAE through multiple accessible channels. These include presentations at local and regional symposia and conferences, where healthcare professionals, policymakers, and academics are present. In addition, findings will be shared through peer-reviewed publications and, where appropriate, summarized in formats suitable for non-academic audiences. Local collaborators are also engaged in communicating key results to relevant stakeholders within their professional networks, ensuring that the findings are accessible, understandable, and relevant to the community*

**Non-human subjects research using specimens/ animals collected as part of the study, or those housed in archival collections. Examples include archaeology, paleontology, botany and zoology.**

**Did the permission you obtained from a local authority to perform the study include an agreement on access to outputs and benefit sharing? This may include procedures to enable fair distribution of the benefits and resources arising from the research performed. Please include any details of Prior Informed Consent and Benefit Sharing Agreements obtained. These may be required by field-specific regulations, for example the Convention on Biological Diversity (CBD) and the associated Nagoya Protocol.**

Not applicable

**If the material used in your study was imported, please A) provide the year it was imported and B) indicate whether permits were obtained to import/export the materials used, C) provide details of any permits obtained. If this information is not available, please indicate th**is.

Not applicable

**If you used archival specimens, please state how the material used in your study was acquired by the institute it is held in and provide details of any permits obtained for the original excavations/ sample collection. If this information is not available, please indicate th**is.

Not applicable

**How was the potential cultural significance of the materials collected in your study to local communities considered in your research design? Were Indigenous peoples and/or local researchers and institutions involved with archaeological excavations / collection of specimens? If so, please provide a description of their involvement.**

*The potential cultural significance of the materials collected was carefully considered throughout the research design. The study primarily involved the collection of survey/interview data related to health-related behaviors, which did not include culturally sensitive artifacts or biological samples. Nevertheless, UAE-based collaborators played a central role in ensuring that all study materials, including questionnaires and data collection tools, were culturally appropriate, respectful, and relevant to the local context.*

*Particular attention was given to the wording of questions, avoidance of sensitive or potentially stigmatizing topics, and alignment with local norms and values. Data collection procedures were conducted by locally based researchers familiar with the cultural context, which facilitated respectful engagement with participants. Furthermore, all study procedures were reviewed and approved by relevant institutional and national ethics committees in the UAE, ensuring adherence to ethical and cultural standards.*

If your manuscript includes photographs of human remains please indicate whether authors obtained permission from descendants or affiliated cultural communities to do so.

Not applicable
